# Lipidome analyses reveal radiation induced remodeling of glycerophospholipid unsaturation in lung tumor

**DOI:** 10.3389/fimmu.2024.1470269

**Published:** 2024-10-24

**Authors:** Jingquan He, Qingqing Yuan, Song Gao, Yue Wang, Haigen Lai, Kaiting Wang, Xiaoman Zhou, Zicheng Zhang

**Affiliations:** ^1^ Department of Radiotherapy, Shenzhen Traditional Chinese Medicine Hospital, The Forth Clinical Medical College, Guangzhou Traditional Chinese Medicine University, Shenzhen, China; ^2^ Department of Radiation Oncology, National Cancer Center, National Clinical Research Center for Cancer, Cancer Hospital & Shenzhen Hospital, Chinese Academy of Medical Sciences and Peking Union Medical College, Shenzhen, China; ^3^ Department of Radiotherapy, Shenzhen Luohu People’s Hospital, Shenzhen, China

**Keywords:** lung cancer, radiotherapy, lipidomics, glycerophospholipids, lipid unsaturation

## Abstract

Radiotherapy is a pivotal treatment for lung cancer, significantly impacting tumor control and patient quality of life. Despite its benefits, the molecular mechanisms underlying radiotherapy-induced biological alterations in lung cancer cells remain inadequately understood. In this study, we employed a mass spectrometry-based lipidomics approach to investigate lipid profile changes in a lung cancer mouse model post-radiation. Lewis lung carcinoma (LLC) cells were injected into C57BL/6J mice, followed by radiation treatment with varying split doses. Our results showed an increase in sterol lipids and a decrease in glycerolipids, specifically triacylglycerides, indicating disrupted lipid storage. Additionally, we observed significant changes in glycerophospholipid unsaturation, suggesting a remodeling of membrane properties that may influence cell survival. Linear regression analysis demonstrated a significant negative correlation between glycerophospholipid unsaturation index and tumor weight, indicating a potential role in radiation-induced tumor cell death. These findings provide new insights into the lipid metabolic pathways affected by radiotherapy and could inform the development of improved therapeutic strategies for lung cancer treatment.

## Introduction

Lung cancer affects millions people every year and is the leading cause of cancer related death worldwide (1). Recently, significant progress has been made in understanding the molecular mechanisms of tumorigenesis, discovering new biomarkers, and developing new treatment methods (2, 3). However, these advancements are still insufficient to cure lung cancer, and the 5-year survival rate for these patients remains very low (4).

Radiotherapy is one of the most important tools for the clinical treatment of lung cancer. Reports indicate that about 50% of lung cancer patients receive and benefit from radiotherapy (5, 6). Although radiotherapy provides effective tumor control, symptom relief, and improved quality of life for lung cancer patients at various stages, there is a subset of patients, especially those with advanced non-small cell lung cancers, who may not respond well to radiotherapy (7, 8). Despite ongoing research and novel treatment approaches aimed at improve patient outcomes (9, 10), it is still far from enough to overcome these challenges. One significant reason is the lack of understanding of radiotherapy-induced biological alterations in lung cancer cells.

The DNA damage response is the most extensively studied signal transduction pathway, providing valuable insights into the underlying molecular mechanisms of tumor cell death induced by radiation (10, 11). Additionally, other pathways such as mTOR signaling pathway mediated protein biosynthesis, VEGF-induced blood vessel biogenesis, and immune changes have been implicated in radiotherapy induced biological alterations (12). Small molecule metabolism remodeling, including glycolysis, cardiolipin metabolism, glutamine biosynthesis, and purine biosynthesis, has also been found to be critical for radiotherapy responses in cancer cells (13–16). However, due to the lack of tumor samples from patients who received radiotherapy, there’s no sufficient information about the global metabolic alterations in tumor cells post-radiation.

In the current study, we treated tumors with radiation in a lung cancer mouse model, and applied a mass spectrometer-based lipidomics approach to investigate lipid profile alterations in tumor cells. We revealed lipid expression changes in tumor cells after radiotherapy, particularly in membrane glycerophospholipids. These data provide new insights into radiation-induced lipid remodeling, which could help in understanding the mechanism of radiation-induced tumor cell death and radio-resistance.

## Materials and methods

### Cell culture

Lewis lung carcinoma (LLC) cells were obtained from National Collection of Authenticated Cell Cultures in China (Shanghai, China) and cultured in DMEM (Dulbecco’s Modified Eagle Medium, Gibco, MA, USA) supplemented with 10% FBS (Fetal Bovin Serum, Gibco) and 100 U/mL Penicillin and 100 μg/mL Streptomycin (Gibco).

### Mouse tumor model

All animal experiments were conducted in accordance with the Animal Welfare Committee at Guangzhou Traditional Chinese Medical University. C57BL/6J male mice weighing approximately 20 g were purchased from Vitalstar Biotechnology (Beijing, China) and maintained on a regular dark-light cycle with free access to food and water. LLC cells were trypsinized and washed with Dulbecco’s phosphate-buffered saline (DPBS, Gibco). After centrifugation at 1000 g for 5 min at room temperature, cells were collected and diluted to a final concentration of 1 X 10^7^/mL. The right flanks of mice were subcutaneously inoculated with 1 million LLC cells.

### Radiation treatment

Mice were randomly divided into four groups 10 days after inoculation, and the tumors were radiation treated with 6 MeV electron rays in different split doses under 2.5% isoflurane. The control group mice received no radiation. The 3.6 Gy group mice were treated with 3.6 Gy (Gray) X 21 times, totaling approximately 103 Gy biological effective dose (BED). The 7.2 Gy Group mice were treated with 7.2 Gy X 9 times (BED≈110 Gy). The 14.4 Gy group mice were treated with 14.4 Gy X 3 times (BED≈105 Gy). All the radiation treatments were conducted by using Elekta Infinity linear accelerator (Elekta, Stockholm, Sweden). Forty-eight hours after radiation, the mice were sacrificed under anesthesia, and tumors were collected, weighted, and frozen immediately in liquid nitrogen.

### Lipid extraction

Twenty milligrams of tumors from each sample were grinded in liquid nitrogen, and lipids were extracted in a buffer containing tert-Butyl methyl ether (MTBE, CNW technologies, Germany) and methanol (MeOH, CNW technologies) (v: v = 5: 1) and SPLASH LIPIDOMIX Mass Spec standard (Avanti Polar Lipids, AL, USA). After a brief sonication, samples were centrifuged at 3000 rpm for 15 min in 4 °C. The supernatant was collected, and an equal volume of MTBE was added. Sonication was then repeated twice, and the supernatant was dried by using a vacuum dryer. Samples were then reconstituted under 10 min sonication in a solution containing Dichloromethane (DCM, CNW technologies), MeOH, and water (v: v: v = 60: 30: 4.5). The quality control (QC) sample was prepared by mixing a small and equal aliquots from all samples together. All procedures were carried out on ice.

### LC-MS lipidomics data acquisition

Samples were analyzed by using an ACQUITY Premier (Waters, MA, USA) ultra-high performance liquid chromatography (UHPLC) system coupled with a SCIEX Triple Quad™ 6500+ mass spectrometer (SCIEX, MA, USA). An ACQUITY UPLC HSS T3 column (Waters) was utilized for UHPLC separation. The mobile phase A consisted of 40% water and 60% acetonitrile, supplemented with 10 mM/L ammonium formate. Mobile phase B consisted of 10% acetonitrile, 90% isopropanol, and 10 mM/L ammonium formate. The elution gradient was set as follows: 80% A at 0min, 40% A at 4min, 2% A at 14 min, 80% A at 16.01 min, and 80% A at 18 min. The flow rate was 0.3 mL/min. The column temperature was maintained at 45°C. The temperature of auto-sampler was set to 10°C. The injection volume was 2 μL per sample.

The ion source parameters for Triple QuadTM 6500+ mass spectrometer were as follows: ion spray voltage: +5500/-4500 V, curtain gas: 40 psi, temperature: 350°C, ion source gas 1: 50 psi, ion source gas 2: 50 psi, and DP: ±80 V.

### Lipidomics data analysis

To identify and quantify compounds, Biobud software (v2.1.4.1, Biotree, Shanghai, China) was used. All peaks were extracted and the peak area was calculated. Peaks with a relative standard deviation (RSD) > 30% across all samples were filtered out. Peaks with more than 50% empty values were further removed. Missing values were filled by multiplying the minimum value by a random number between 0.1 and 0.5. After all these steps, 700 peaks were retained.

### Quantitative RT-PCR analysis

Total RNAs of tumor samples were prepared by using Trizol reagent (Thermofisher Scientific, MA, USA). 1 μg RNAs from each sample were reverse transcribed to cDNA by using SuperScript™ IV reverse transcriptase (Thermofisher Scientific). The levels of glycerophospholipid metabolism related genes were tested by using SYBR green Real-time PCR mix (Thermofisher Scientific) in QuantStudio™ 5 system (Thermofisher Scientific). The mRNA expression level was quantified and normalized to that of GAPDH by using 2^-ΔΔCT^ (17). The primers used were as follows: Agpat3, forward: 5’-CTGCTTGCCTACCTGAAGACC-3’, reverse: 5’-GATACGGCGGTATAGGTGCTT-3’; Plpp1, forward: 5’-AGTCTCAGCTAGTCAGTCCTTGA, reverse: GGCTTGAAGATAAAGTGCGACAA; Pld1, forward: 5’-TCGTTTTGTGGACTGAGAACAC-3’, reverse: 5’-GCTGCTGTTGAAACCCAAATC-3’; Scd4, forward: 5’-GCCCACTTGCCACAAGAGAT-3’, reverse: 5’-GTAGCTGGGGTCATACAGATCA-3’; Gpd1, forward: 5’-ATGGCTGGCAAGAAAGTCTG-3’, reverse: 5’-CGTGCTGAGTGTTGATGATCT-3’; Gpcpd1, forward: 5’-TGCCAACACAGGGATGGAGTA-3’, reverse: 5’-TGCTTCTGCCGAACCATTGTA-3’.

### Statistical analysis

Statistical analysis was performed by using IBM SPSS statistics (IBM, NY, USA), Microsoft Excel (Microsoft, WA, USA) and the R language (R Foundation, Vienna, Austria). The lipid expression profile was depicted by principal component analysis (PCA) and partial least-squares discriminant analysis (PLS-DA). Significantly changed lipids were identified by orthogonal projections to latent structure-discriminant analysis (OPLS-DA) and student’s t test. Lipids with a variable importance in projection (VIP) > 1 in OPLS-DA analysis and p-value < 0.05 in student’s t test were considered as significantly changed lipids. Significantly altered lipid classes in response to radiation were assessed by Fisher’s exact test. For multi-group comparisons, one-way ANOVA or two-way ANOVA tests were used. For the relationship between glycerophospholipid unsaturation and tumor weight, linear regression analysis was applied. A p-value < 0.05 was considered significant, and data were presented as mean ± standard error (mean ± SE).

## Results

### Radiation reduces tumor growth

**Figure 1:**
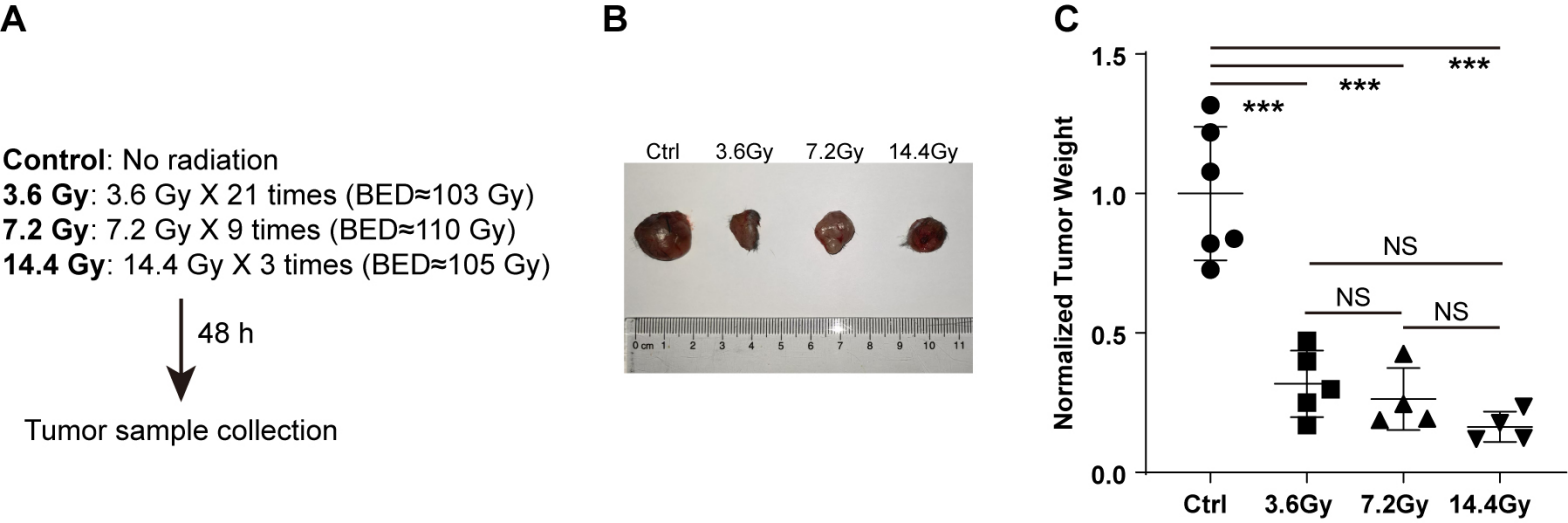
Radiation reduces tumor weight. **(A)** Different split doses were continuously delivered to LLC tumors once a day. Forty-eight hours after similar total biological effective doses fully delivered, tumor samples were collected. **(B)** Representative images to show tumor size alterations after radiation treatment. **(C)** Tumor weight changes after radiation treatment. NS, not significant; ***p-value < 0.001 by one-way ANOVA followed by holm-sidak test.

To determine the influence of radiation on tumor growth, the right flanks of mice were injected with LLC cells. All mice started to develop tumors at about one week after inoculation. The mice were randomly divided into four groups: control, 3.6 Gy, 7.2 Gy and 14.4 Gy, which were administrated with different split doses of radiation starting 10 days after injection. Except for the control group, which didn’t receive radiation, all other groups received similar biological effective doses (BED) (**Figure 1A**). Two days after all radiation doses were fully administered, the mice were sacrificed and the tumors were collected. The weight of tumors that received radiation was significantly reduced compared to control mice (**Figure 1B**). This reduction is mostly dependent on the BED the tumors received and is not related to the split dose, as there is no significant difference in tumor weight among the 3.6 Gy, 7.2 Gy and 14.4 Gy groups (**Figure 1B**).

### Radiation alters lipid profile in tumors

**Figure 2:**
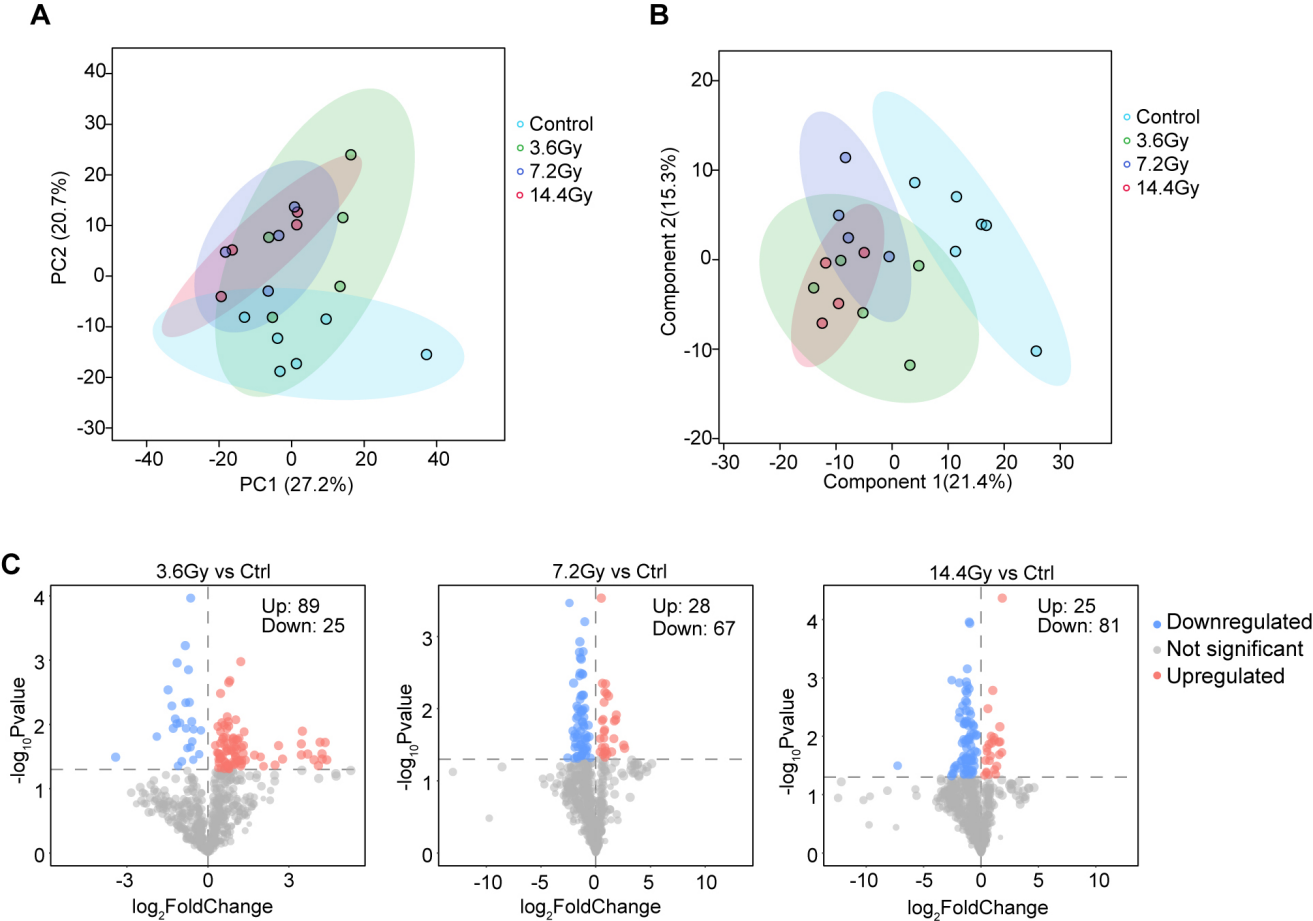
Radiotherapy alters lipid expression profiles in LLC tumors. **(A)** PCA analysis showed clear separation between the control group and radiation groups. No clear separation was observed among three radiation groups. **(B)** PLS-DA analysis showed clear separation between the control group and radiation groups. No clear separation was observed among three radiation groups. **(C)** Volcano plots showed the alterations of lipids after radiation treatment. Red indicates significantly upregulated lipids; blue indicates significantly downregulated lipids. The number of significantly changed lipids is shown in the diagram.

Few studies have reported the impact of radiation on cancer cell lipid metabolism. Therefore, we performed a LC-MS lipid profiling study in LLC tumors to uncover the global lipidome alterations after radiation. A total of 700 lipid species were identified and quantified. PCA analysis, based on all identified lipids, showed a clear separation between the control group and all radiation groups. However, no obvious separation among the three radiation groups was observed (**Figure 2A**). To confirm this, we further conducted PLS-DA analysis, and similar results were obtained (**Figure 2B**). These data suggest that the lipid expression profile was significantly changed in tumor cells in response to radiation, and it was not related to the split doses administered. We thus focused on the differential lipid expression analysis in radiation groups against control group. We found that radiation induced significant expression changes of many lipids (VIP > 1 and p-value < 0.05), regardless of the split doses (**Figure 2C**). These observations indicate a global impact of radiation on tumor cell lipid expression.

### Alterations in lipid storage due to radiation

**Figure 3:**
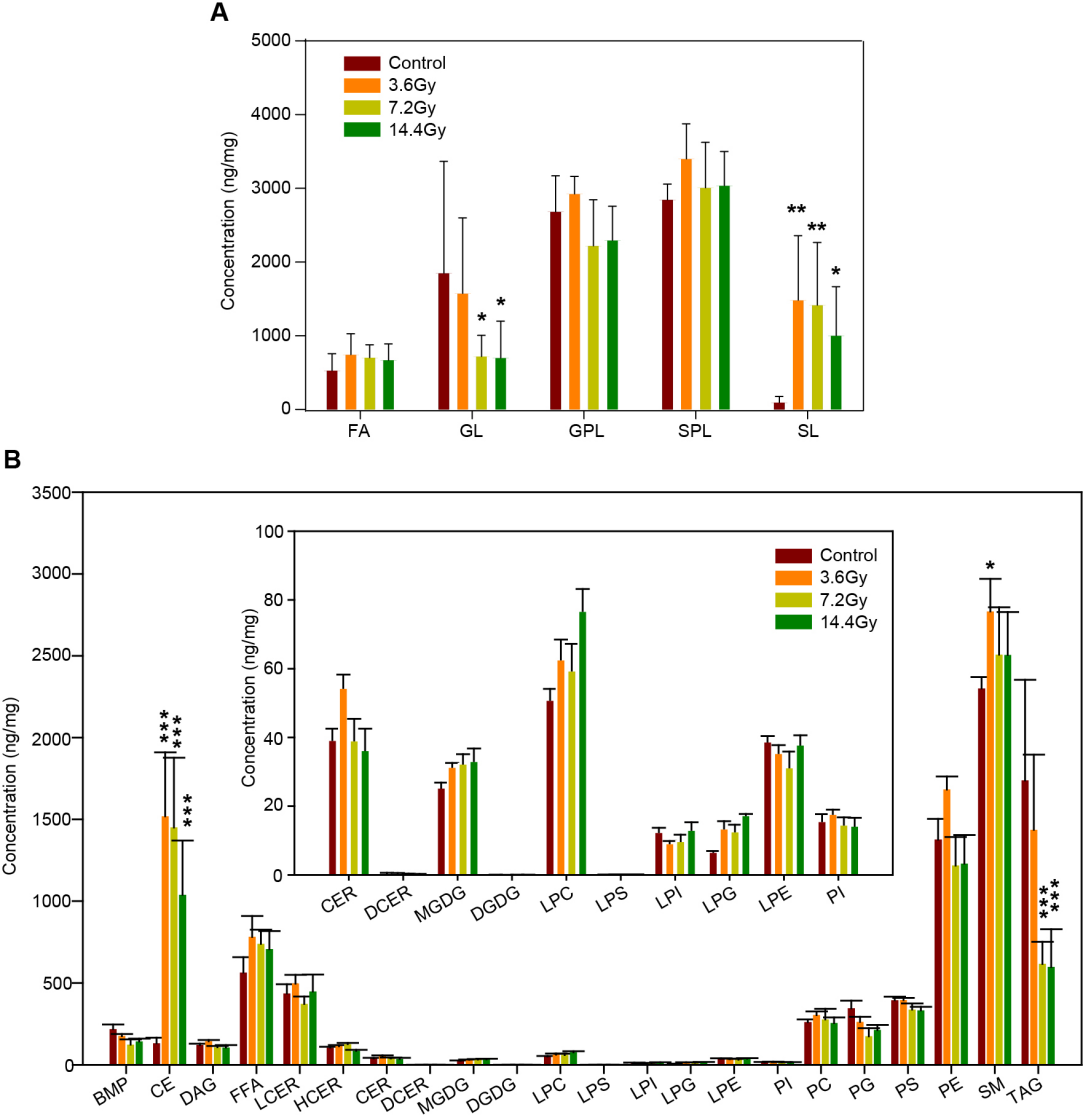
Impact of radiation on different lipid classes. **(A)** The alteration of the summed total concentration of lipids at the class level after radiation. GL, glycerolipids; SL, sterol lipids. *p-value < 0.05; **p-value < 0.01 by two-way ANOVA. **(B)** The total concentration of lipids at the sub-class level in response to radiation. Insert, the content of several low abundance lipids at the sub-class level. CE, cholesterol esters; TAG, triacylglycerides. ***p-value < 0.001 by two-way ANOVA.

According to lipid classification criteria, all the 700 lipid species were divided into several classes: fatty acyls (FA), glycerolipids (GL), glycerophospholipids (GPL), sterol lipids (SL) and sphingolipids (SPL). To compare the changes in lipid class, the abundance of all lipid species in each class were summarized. Statistical analysis revealed that the level of total SL was significantly elevated in response to radiation in the 3.6 Gy, 7.2 Gy, and 14.4 Gy groups (**Figure 3A**). In contrast, the level of total GL was significantly reduced in the 7.2 Gy and 14.4 Gy groups (**Figure 3A**).

To further understand the influences of radiation on lipid metabolism, all lipids were further divided into sub-classes. For example, GL was divided into diglyceride (DAG), triglyceride (TAG), monogalactosyl diglyceride (MGDG), and digalactosyl diglyceride (DGDG). Interestingly, there were no alterations in DAG, MGDG, and DGDG, but the total level of TAG was significantly decreased in response to radiation (**Figure 3B**). Together with the increase of total cholesterol esters (CE) (**Figure 3B**), these data demonstrate the impact of radiation on lipid storage in tumor cells.

### Radiation affects glycerophospholipids metabolism

**Figure 4:**
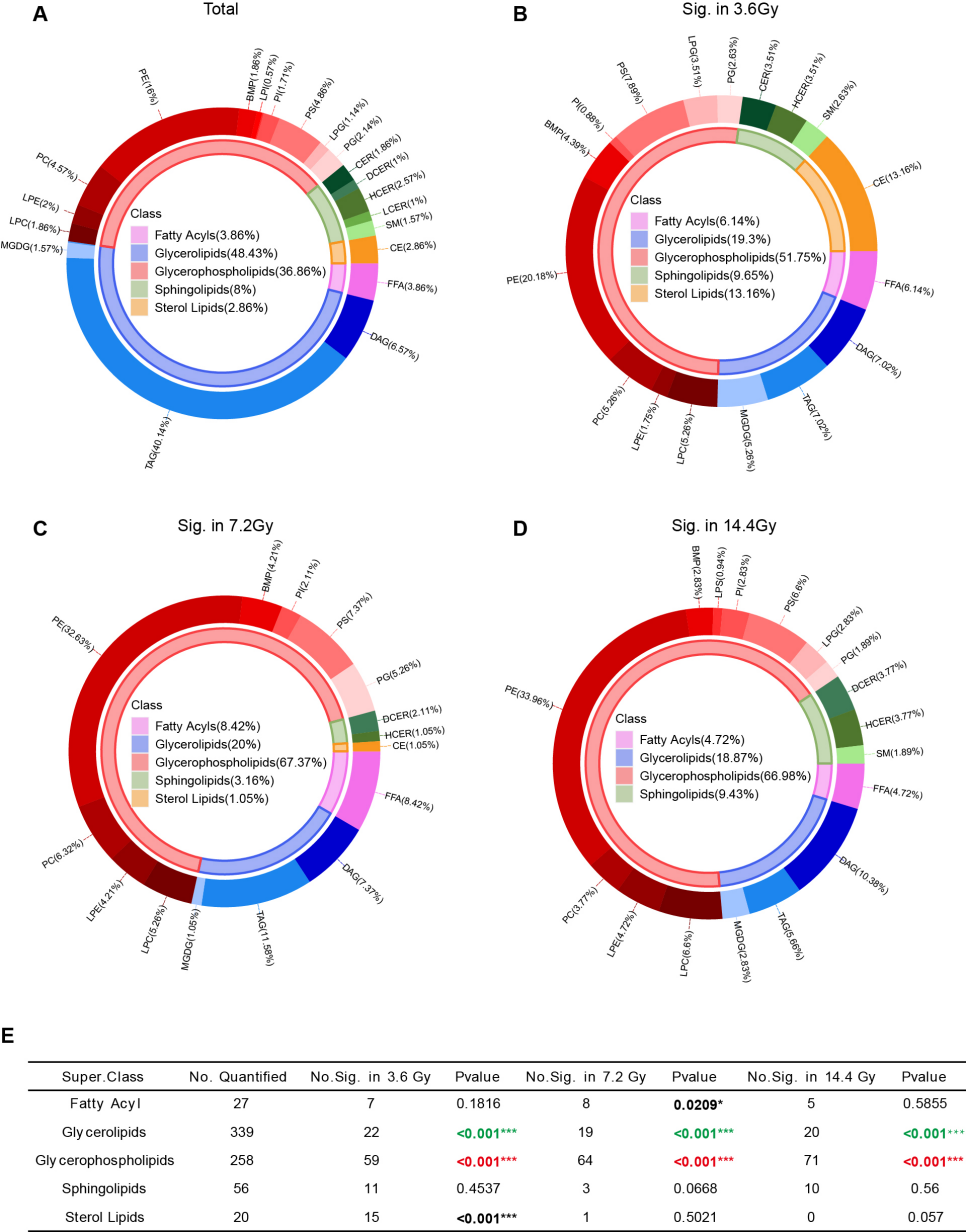
Lipid class composition changes after radiation exposure. **(A)** Pie graph of the lipid composition according to the number of lipids identified. The inner circle represents lipid class composition, and the outer circle represents lipid composition at the sub-class level. **(B)** Pie graph of the lipid composition of significantly altered lipids in the 3.6 Gy group. **(C)** Pie graph of the lipid composition of significantly altered lipids in the 7.2 Gy group. **(D)** Pie graph of the lipid composition of significantly altered lipids in the 14.4 Gy group. **(E)** Enrichment analysis of significantly changed lipids at the class level. *p-value < 0.05, ***p-value < 0.001 by Fisher’s exact test. Red, significantly enriched; Green, very small portion significantly changed.

Among all of the identified lipids, GL accounts for majority, which is about 48.43% (339 out of 700). GPL accounts for 36.86% (258 out of 700) (**Figure 4A**). Radiation treatment altered the abundance of many lipids. Specifically, 114 lipids were significantly changed in the 3.6 Gy group, 95 lipids in the 7.2 Gy group, and 106 lipids in the 14.4 Gy group (**Figure 2C**). Among all significantly changed lipids, GPL accounted for a large portion in all three radiation groups: 51.75% in the 3.6 Gy group, 67.37% in the 7.2 Gy group, and 66.98% in the 14.4 Gy group (**Figures 4B–D**). Further analysis using Fisher’s exact test revealed a significant enrichment of GPL among the significantly altered lipids in all three radiation groups (**Figure 4E**), indicating a preferential effect of radiation on GPL metabolism.

We also noticed that very small portion of GLs was significantly changed after radiation (**Figure 4E**), suggesting minimal influence of radiation on GL metabolism.

### Membrane glycerophospholipid unsaturation remodeling in radiation

**Figure 5:**
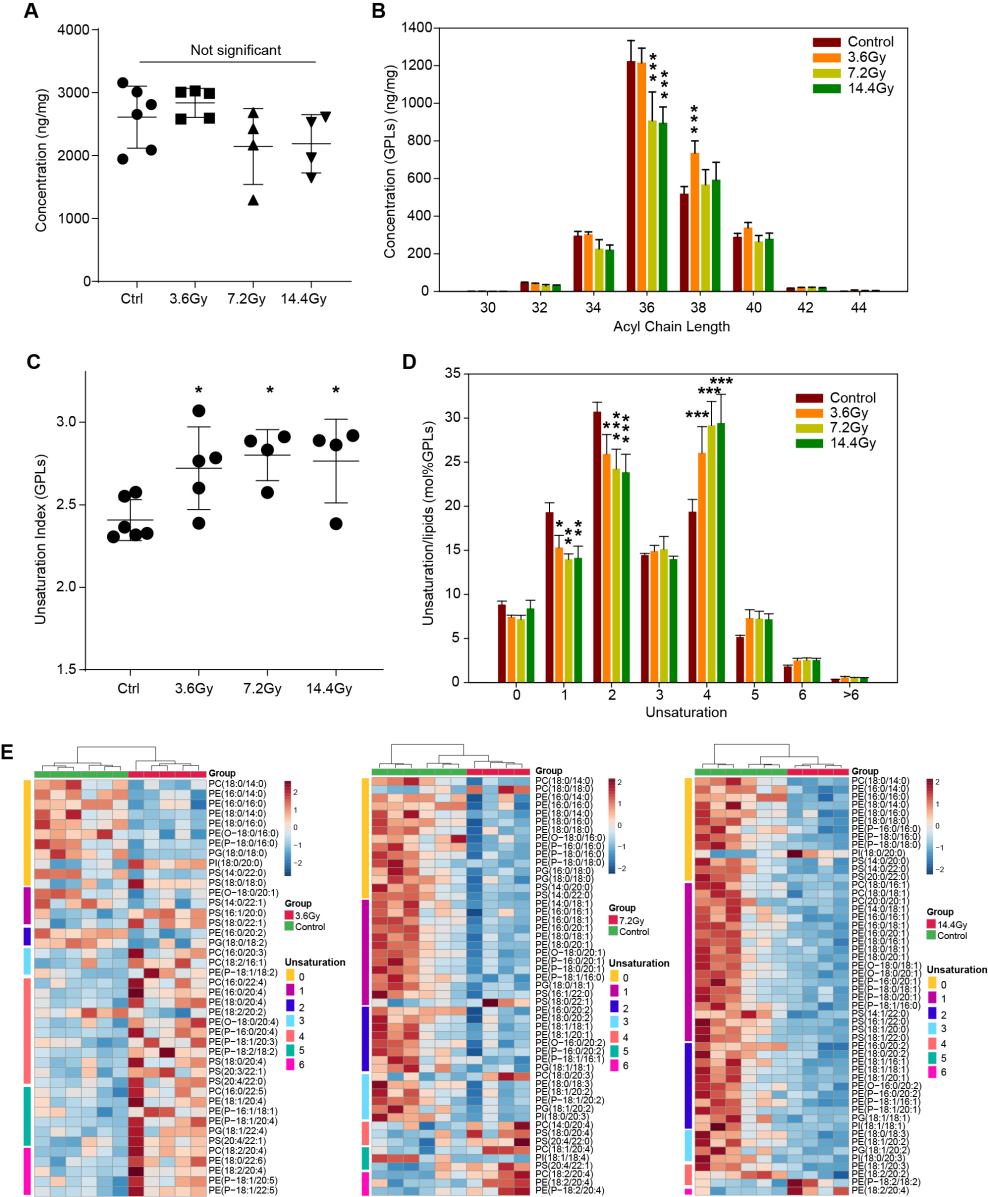
Glycerophospholipid unsaturation remodeling in LLC tumors after radiation treatment. **(A)** The summed amount of all identified glycerophospholipids in different groups. No significant alteration was observed by one-way ANOVA. **(B)** The concentration changes of glycerophospholipids with different acyl chain lengths in response to radiation. ***p-value < 0.001 compared to control group by two-way ANOVA followed by holm-sidak test. **(C)** The unsaturation index (concentration weighted average lipid unsaturation) changes of all glycerophospholipids quantified in response to radiation. *, p-value < 0.05 compared to control group by one-way ANOVA followed by holm-sidak test. **(D)** Remodeling of glycerophospholipids unsaturation induced by radiation. Significant decreases in glycerophospholipids containing one and two unsaturations, and significant increases glycerophospholipids containing four unsaturations were observed. *p-value < 0.05; **p-value < 0.01 and ***p-value < 0.001 compared to control group by two-way ANOVA followed by holm-sidak test. **(E)** Heatmap of all significantly changed glycerophospholipids in the three radiation groups. The unsaturation of these lipids is indicated on the left side of each plot.

The composition and properties of GPLs are important for cell membrane structure and transmembrane signaling. Thus, we analyzed the property alterations of GPLs in response to radiation. There were no significant changes in total GPL in any of the three radiation groups (**Figure 5A**). However, we observed a decrease of long acyl chain-containing GPLs after radiation, particularly GPLs containing 36 carbons in the 7.2 Gy and 14.4 Gy groups (**Figure 5B**). Conversely, 38 carbons containing GPLs was observed significantly increased in the 3.6 Gy group (**Figure 5B**).

In addition, a significant increase in unsaturation in GPLs was observed in all three radiation treatment groups (**Figure 5C**). This is most likely due to the significant decrease in GPLs with one and two unsaturations and the significant increase in GPLs with four unsaturations (**Figure 5D**). To fully understand the GPL alterations, we depicted all the significantly changed GPLs by using a heatmap (**Figure 5E**). In the 3.6 Gy group, saturated GPLs were significantly decreased, and unsaturated GPLs were increased. Interestingly, in the 7.2 Gy group, GPLs containing more than 4 unsaturations were significantly increased, while others were significantly decreased. In the 14.4 Gy group, only GPLs containing more than 5 unsaturations were increased. These data suggest a differential influence of different split radiation doses on GPL acyl chain length and unsaturation.

### Radiation alters GPL metabolism related genes

**Figure 6:**
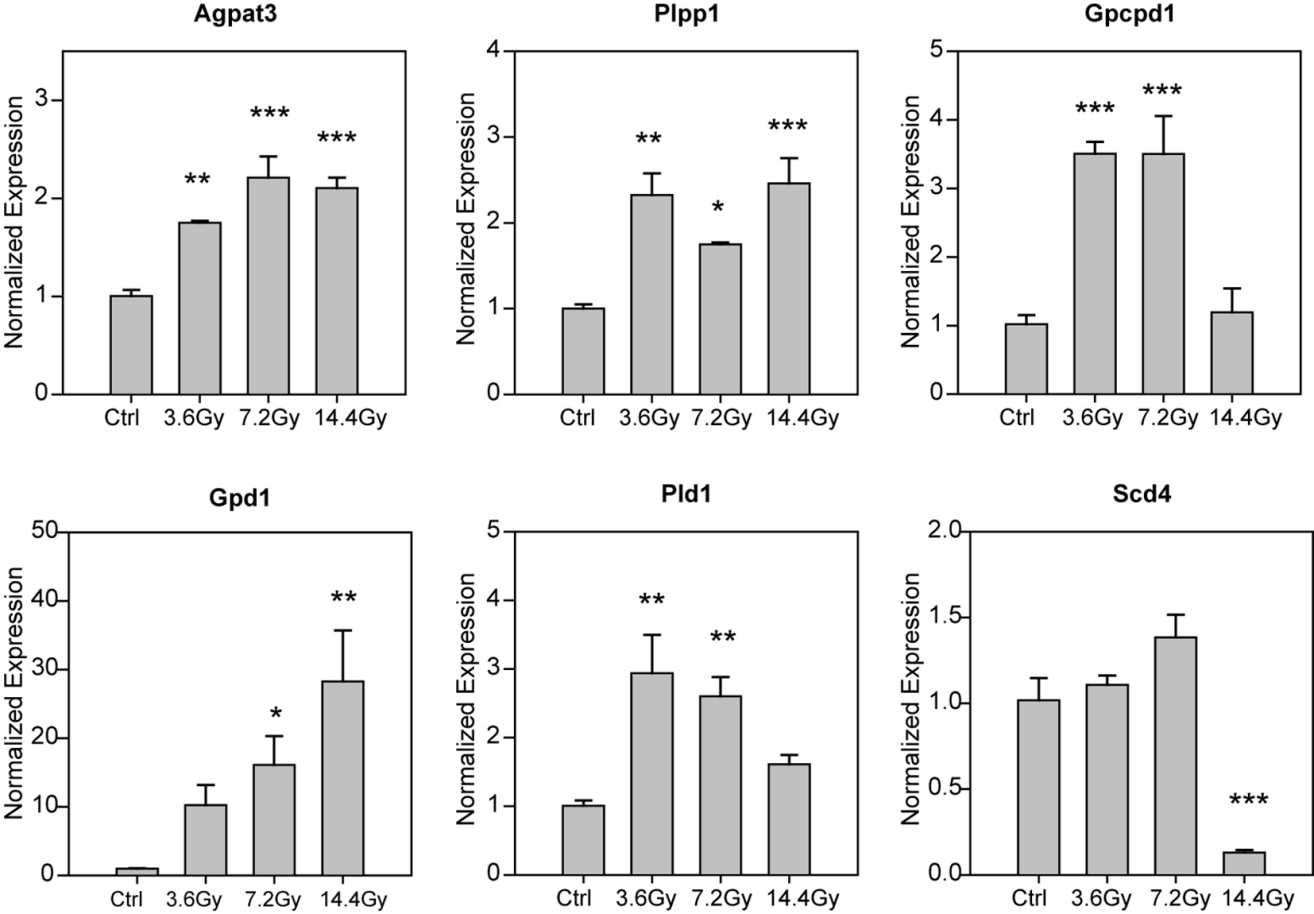
Alteration of glycerophospholipid metabolism related genes. The mRNA expression level of glycerophospholipid metabolism related genes were measured by using QPCR, and the statistical analysis results were showed. *p-value < 0.05; **p-value < 0.01 and ***p-value < 0.001 compared to control group by one-way ANOVA followed by holm-sidak test.

Since radiation significantly altered tumor glycerophospholipid composition, we thus checked the expression of glycerophospholipid metabolism related genes by QPCR. We found that several genes were significantly changed (**Figure 6**), including Agpat3, Plpp1, Gpcpd1, Gpd1, Pld1 and Scd4. Among them, Scd4 was found to be significantly reduced only in 14.4 Gy group, others were elevated either in two groups or in all three radiation groups. Interestingly, Pld1 and Gpd1 have be reported to be critical for cancer progression and metastasis (18, 19). These data further demonstrate the influence of radiation on glycerophospholipid metabolism.

### Correlation of tumor weight and GPL unsaturation

**Figure 7:**
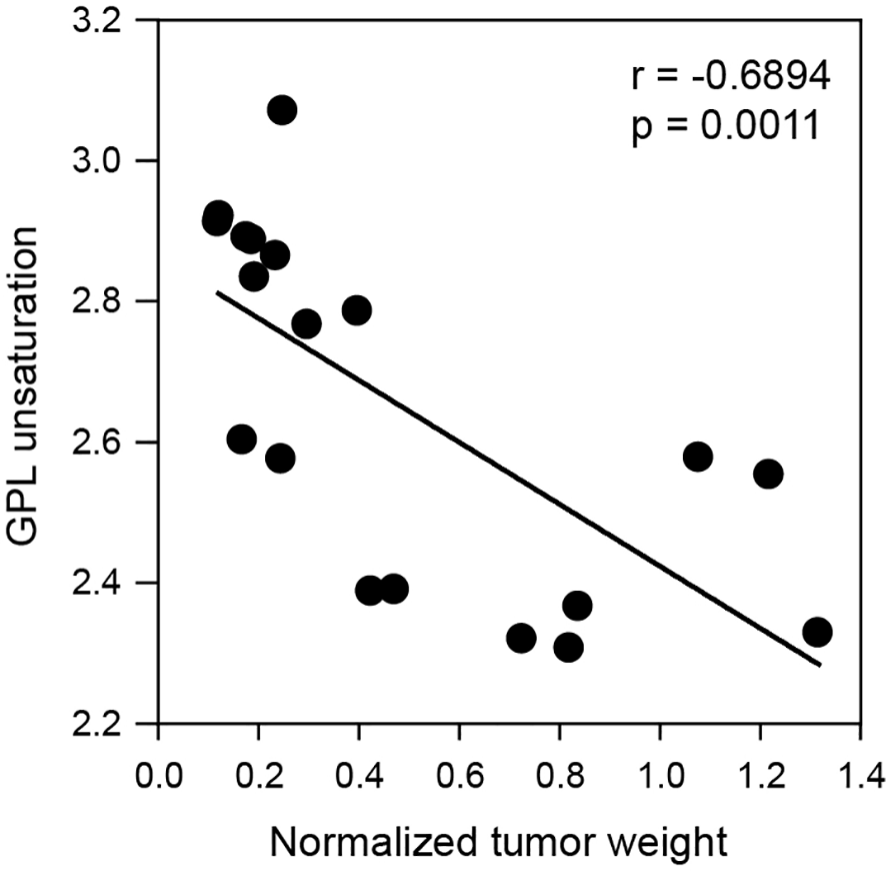
Correlation of glycerophospholipid unsaturation to tumor weight. A linear regression model demonstrates glycerophospholipid unsaturation index is significantly and negatively correlated with tumor weight. The p-value and correlation coefficient are shown in the plot.

Cell membrane structural alterations that induce membrane permeabilization is a key phenotype of cells undergoing death (20). Thus, we investigated whether GPL unsaturation alterations due to radiation are correlated with tumor cell death. We analyzed the relationship between tumor weight and the GPL unsaturation index by using linear regression. As shown in **Figure 7**, GPL unsaturation is negatively correlated with tumor weight, with a correlation coefficient of -0.6894. These data suggest that radiation induced changes in GPL unsaturation may contribute to lung tumor cell death.

## Discussion

In this study, we applied a LC-MS lipidomics approach to investigate radiation induced lipidome alterations in a lung cancer mouse model. We found that the lipid expression profile of cancer cells exposed to radiation is markedly different from that of non-radiated cells. Additionally, we observed alterations in lipid storage and changes in the glycerophospholipid composition after radiation. These data suggest that lipid metabolism and lipid structures are key targets of radiotherapy, which may be critical in radiation induced cancer cell death.

Lipids are key structural and signaling molecules in cells. Several studies have done to uncover lipid alterations post-radiation either in plasma, in cultured cells (21, 22), or in normal organs (23–25), while rare studies were conducted in tumor samples. In our study, we carried out lipidomics analysis in lung tumors, which could be more accurate to reflect the changes inside the tumor cells undergoing radiotherapy. Besides, our data showed that lipids classes, such as glycerophospholipids and sphingolipids, are significantly altered in response to radiotherapy, which is similar to previous studies (21, 22). Most importantly, we observed significant increase of glycerophospholipid unsaturation in tumor sample post-radiation, suggesting a profound impact on membrane properties and a potential influence of cellular signaling.

Different dose deliveries of radiation therapy are widely used in clinical practice (26). Both low-dose fraction and high-dose fraction radiotherapy have similar benefits and risks, but may have different molecular mechanisms (27, 28). For example, DNA damage response, oxidative stress, cell death pathways, and the triggering of immune responses, are similar biological mechanisms underlying different doses (26, 29). However, the effect on cell cycle redistribution and regeneration of hypoxic tumors may be varying between different doses (30, 31). In our study, we observed similar tumor weight decrease in the 3.6 Gy, 7.2 Gy and 14.4 Gy groups (**Figure 1**), suggesting similar benefits of different split dose radiations on LLC tumors. Additionally, we also observed similar effects of different split doses on lipid compositions, including impact on sterol lipid abundance and increase of unsaturation of glycerophospholipids (**Figures 4E**, **5C**). In contrast, differences were also observed, such as different impact on total triacylglycerol abundance (**Figure 3**). Additionally, we also observed the varying effects of different split doses on the fatty acid chain composition of glycerophospholipids (**Figure 5E**). These data suggest the underlying mechanism that affect similar tumor reduction in different radiation groups could be different.

Neutral lipids, including triacylglycerides (TAG) and cholesterol esters (CE), are mainly stored in lipid droplets. They play a central role in lipid metabolism for energy storage and membrane biosynthesis and are crucial in carcinogenesis (32). Our data showed that radiation affected the levels of neutral lipids (**Figure 3**). Specifically, cholesterol esters were significantly increased (about 10-fold) in lung cancer cells in all three radiation groups. This aberrant cholesterol storage may contribute to the radio-resistance of cancer cells (33), facilitating tumor recurrence. Additionally, the release of cholesterol from cholesterol esters after radiation could be a potential risk factor for radiation-induced heart disease (34). In contrast, the abundance of TAG was significantly reduced in the 7.2 Gy and 14.4 Gy groups, but no significant difference was observed in the 3.6 Gy group, suggesting a fraction dose specific impact. A previous study reported that TAG can be lipolyzed to form free fatty acids and fuel mitochondrial fatty acid oxidation to provide energy for cell proliferation (35). Thus, our study suggests that high-dose fraction treated tumors may be less resistant to radiation due to decreased TAG content.

Glycerophospholipids, key structural components of cell membrane, are critical determinants of membrane properties and are essential for transmembrane signaling (36). During cell death, whether by apoptosis or necrosis, the cell membrane undergoes disruption and lose of integrity (20). Besides, glycerophospholipid metabolism is also critical for tumor growth and survival (37), tumor metastasis (38), and tumor survival (35). However, it is not well understood how membrane alterations correlate with tumor cell death under radiation. We observed that glycerophospholipid undergoes increased unsaturation in response to radiation (**Figure 5**), suggesting an alteration of membrane glycerophospholipid bilayers and a disruption of membrane properties that could impact cell survival during radiation. Further regression analysis confirmed the correlation between GPL unsaturation and tumor cell death (**Figure 7**). These data highlighted the potential role of lipid unsaturation status in radiation induced tumor cell death, suggesting the clinical utility by modulating lipid unsaturation in tumor radiotherapy.

In this study, we analyzed lipidomic profile alterations in a lung tumor model in response to radiation. We revealed changes in lipid storage and glycerophospholipids and uncovered the possible role of GPL unsaturation in radiation induced tumor cell death. We also investigated the changes of glycerophospholipid metabolic genes in response to radiation. Further analysis regarding the molecular and cellular mechanism of lipid metabolism alterations under radiation and the potential impact on tumor cell death need to be addressed. In addition, an analysis of the normal tissues, and not just the tumors, could provide more information on the systematic effect of radiation. Our data provide new insights into understanding the biological effects of cancer radiotherapy.

## Data Availability

The raw data supporting the conclusions of this article will be made available by the authors, without undue reservation.
